# Feasibility of written instructions in airway management training of laryngeal tube

**DOI:** 10.1186/1757-7241-19-56

**Published:** 2011-10-10

**Authors:** Jouni Kurola, Heikki Paakkonen, Tapio Kettunen, Juha-Pekka Laakso, Jouko Gorski, Tom Silfvast

**Affiliations:** 1 Division of Prehospital Emergency care, Emergency and Intensive Care, Kuopio University Hospital, PO Box 1777, FIN-70210 Kuopio, Finland; 2 Arcada University of Applied Sciences, Jan-Magnus Janssonin aukio 1, FIN-00550 Helsinki, Finland; 3 Emergency Services College, PO Box 1122, FIN-70821 Kuopio, Finland; 4 Department of Anaesthesiology and Intensive Care Medicine, Helsinki University Central Hospital, PO Box 340, FIN-00029 Helsinki, Finland

**Keywords:** Airway management, laryngeal tube, training

## Abstract

**Background:**

Airway management is of essential importance in emergency care. Training and skill retention of endotracheal intubation (ETI) - the technique considered as the "gold standard" -, poses a problem especially among care providers experiencing a low frequency of airway management situations. Therefore, alternative airway devices such as the laryngeal tube (LT) with potentially steeper learning curves have been developed and studied. Our aim was to evaluate in a manikin model the use of LT after no other training than written instructions only.

**Methods:**

To evaluate the amount of training required to use the LT in a scenario of airway compromise, we assessed the feasibility of providing written instructions and pictures showing its use to 67 out- and in-hospital emergency care providers attending an Emergency Care conference. The majority of the participants were either nurses or firemen with a median of 5 years' history of work in emergency care.

**Results:**

In this study 55% of all participants inserted the LT on the first attempt without additional instructions. An additional 42% required verbal instructions before successful insertion. Overall, 97% of the participants successfully inserted the LT with two attempts.

In logistic regression analysis, no relationship was detected between background variables (basic education, experience of emergency work, frequency of bag-valve-mask ventilation (BVM) and frequency of ETI) and successful insertion of the LT in less than 30 seconds, ability to maintain normoventilation (7 l/min) and need for further instructions during the test.

**Conclusions:**

We found that in this pilot study majority of emergency care providers could insert LT with one or two attempts with written instructions, pictures and verbal instruction. This may provide an option to simplify the training of airway management with LT.

## Introduction

Endotracheal intubation (ETI) is considered the "gold standard" for advanced airway management in emergency care, but due to fairly long period of preceding training and difficulties related to the maintenance of skills it is not recommended for prehospital airway management by paramedics [1,2]. On the other hand, also bag - valve mask ventilation (BVM) has been shown to be difficult [3]. Especially in prehospital care the low frequency of airway management situations per individual poses a problem regarding skill maintenance, and therefore other devices showing shorter learning curves and better skill retention have been developed and studied [4].

The laryngeal tube (LT) is a device which can be blindly inserted into the oropharynx of the patient. The disposable LT (LT-D) is single-lumen device which is made from PVC and it has two cuffs, which are inflated with a single syringe [5]. The distal balloon lies in the opening of the oesophagus while the proximal one obstructs the pharynx at the base of the tongue. Between the two cuffs, two openings in the tube allow air to enter the larynx. The device has been successfully used in anaesthesia and also tested in manikin models by clinically inexperienced emergency medical personnel after manikin training only [6-8] and studied in clinical prehospital emergency settings with success [9-11]. It seems to be a device which requires a modest amount of training to insert and use.

The aim of our study was to evaluate how well emergency medical personnel can insert the LT-D and maintain ventilation in a manikin without other prior training than written instructions and photographs depicting the use of this device.

## Methods

The study was conducted at the national Emergency Services College (ESC) in Kuopio, Finland during a conference on Emergency Care for both out- and in-hospital emergency medical personnel. No ethical board approval was applied. Upon registration and during the conference, the delegates (190 altogether) were informed that they had a voluntary chance to test their ventilatory skills using a novel device. No details of the study protocol were revealed at this point, and no inducements were offered. Those willing to participate were guided to a classroom where they were asked to complete a sheet on background information about themselves. Data collected included age, type of work (out-of-hospital/in-hospital), basic education, work history, and previous acquaintance with the LT. Thereafter the participants were given one sheet of paper with details on the LT and step by step instructions on how to use the device. Insertion of the LT-D (Laryngeal Tube-Disposable, VBM Medizintechnik GmbH Sulz, Germany) into an AMBU^® ^Mega Code Trainer (Ambu Corp. Copenhagen, Denmark) was also displayed on eight photographs posted on the classroom wall. One LT-D size 4 was available for examination. No other information or training was given to the participants prior the test.

At the beginning of the test, the LT-D size 4, the syringe for cuff inflation and a bag-valve ventilator (Laerdal Inc. Stavanger, Norway) were ready on a table. Each participant was tested separately and told that he was expected to insert the LT-D in a scenario with a witnessed collapse and apnoea. No prior patient assessment or ventilations were to be performed. The participants were asked to insert the LT-D, inflate the cuffs, verify correct positioning by auscultation, fix the tube and start BVM ventilation aiming at normoventilation. If insertion was unsuccessful or difficult, an instructor could give further advice on how to proceed.

To obtain ventilatory data, a connector for side-stream spirometry (Datex-Ohmeda CS 3, Datex Corp. Helsinki, Finland) had been inserted in the lower part of the trachea of the manikin to measure airway pressures and ventilation volumes. Two independent observers collected the time needed for insertion, starting from the opening of the mouth to the first measurable ventilation in spirometry which was also time point when insertion was called successful. Spirometry values were then collected at 30, 60 and 90 seconds from the beginning of ventilation. Any help requested from the instructors was also recorded.

Results were analysed using the Windows SPSS version 12.0 (SPSS Inc., Chicago, USA) software. Numerical data are presented as median with interquartile range unless stated otherwise. A logistic regression model was fitted to assess explanatory background variables on the successful insertion of the LT-D in less than 30 s, the ability to maintain normoventilation (defined as 7 l/min), and the need for further instructions to insert LT-D.

## Results

**Figure 1:**
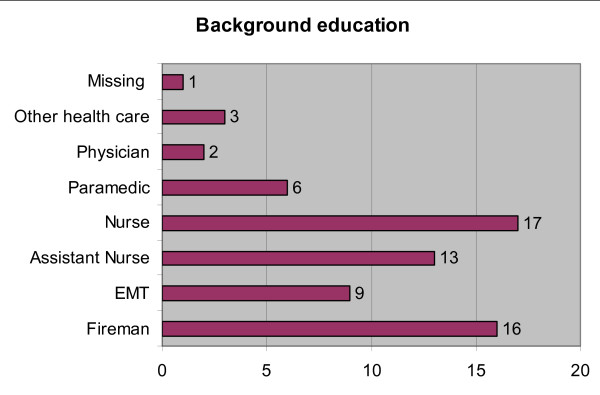
**Background education of the participants (n = 67)**. EMT = Emergency Medical Technician.

A total of 67 conference delegates participated in the test. Their median age was 30 years (27 - 37), and 84% of them were males. Sixty-one per cent presently worked in EMS services and 39% in hospital. The majority of the participants were either nurses (25%) or firemen (23%) (Figure 1). Their experience in emergency care was 5 years (1 - 9). Two participants had previously received training to use the LT but neither had actually used it. Forty participants (60%) reported that they assist ventilation with BVM more than 12 times a year, and 23 (34%) participants estimated that their frequency of ETI was more than 12 times annually. Forty-three participants (64%) reported ETI frequencies once a year or less.

**Figure 2:**
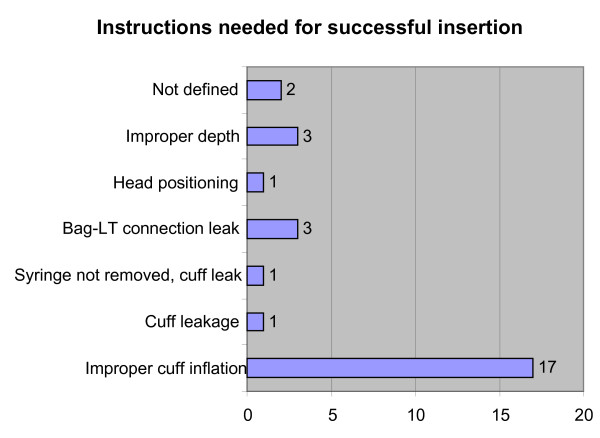
**Instructions needed for successful insertion (n = 28)**. LT = Laryngeal tube.

A total of 65 of the 67 participants (97%) successfully inserted the LT-D. Thirty-seven (57%) of them succeeded at the first attempt and without the need for any other instructions than those provided before the beginning of the test. The need for verbal supplemental instructions before successful insertion among the 28 remaining participants was mostly related to improper cuff inflation (Figure 2). The supplemental instructions involved verbal advice to re-check the issue which appeared to prevent the successful insertion of the LT-D.

The time needed for insertion, measured from the opening of the mouth to the first measurable ventilation in the whole group was 31.5 s (25.0 - 47.3). In the group without a need for instructions it was 28.0 s (23.0 - 34.0), and for those who needed instructions it was 48.0 s (28.0 - 68.0). For the whole group, minute volume ventilation was 6.5 l (5.2 - 8.3) and peak airway pressure 13.6 mmHg (10.7 - 16.5).

In logistic regression analysis, we did not detect any relationship between background variables (background education, emergency work experience, frequency of BVM and frequency of ETI) and the three main variables related to successful use of LT-D (successful insertion in less than 30 seconds, ability to maintain normoventilation (7 l/min) and need for further instructions during insertion).

## Discussion

In this study we found that virtually all participants could insert the LT-D in a manikin after written instructions, but 43% only after verbal assistance, mostly related to improper inflation of the cuff causing air leakage.

The need for alternative airway management devices especially in emergency care is evident. The value of paramedic performed prehospital intubation is undetermined [12], and even highly trained paramedical personnel have been shown to have difficulties with this procedure [13]. Maintaining adequate skills poses a further problem. Also, several unsuccessful intubation attempts increase the risk for complications [14].

Training of airway management in emergency care should consist of didactic lessons and simulation training in manikins. The possibility of prehospital staff to rehearse on anaesthetised patients in the operating room is often limited. In rural areas the low frequency of patients requiring emergency airway management poses a huge challenge for the prehospital care provider's skill retention. In previous studies the LT has been found relatively easy to use after manikin training only [8,15,16]. The present study suggests that the training required to use this device with written instructions and additional verbal guidance is effective. It seems, however, that during training with this device attention should be focused especially to avoid improper cuff inflation causing air leakage and on the proper depth of insertion. Therefore training completely without professional instructor is not recommended. The time needed for successful insertion and beginning of ventilation was comparable to that reported in other studies using a manikin [8,15,16]

Some obvious limitations in the interpretation of these results should be kept in mind. The fact that all participants had at least some experience of emergency work may be of importance. It is possible that these individuals require a shorter training with new airway devices compared to inexperienced students. Still, two thirds of the participants reported frequencies of ETI less than once a year, which obviously is too low for gaining experience or maintaining skills in emergency airway management. Another consideration is that the participants in the study may have been better motivated or in another way more talented, and thus created a selection bias which positively affected the results. Also, the simulated scenario did not require normal patient assessment and the stress caused by a live situation was absent, factors which obviously would influence the performance of the care provider in real life [17].

## Conclusions

In this study 97% of participants were able to insert the LT-D and from those who succeeded, 57% on the first attempt after written instructions and pictures only. The rest (43%) required verbal instructions before successful insertion and ventilation. Although the use of the LT-D seems to require minimal training, attention should be focused on training of correct depth of insertion and cuff inflation.

## Competing interests

The authors declare that they have no competing interests.

## Authors' contributions

All authors read and approved the final manuscript. JK, HP and TS designed the study. HP, TK, JPL, JK and JG performed the study. JK, HP and TS prepared the manuscript. JK and JG made statistical analysis.

## References

[B1] Bradley JS Billows GL Olinger ML Boha SP Cordell WH Nelson DR Prehospital oral endotracheal intubation by rural basic emergency medical technicians Ann Emerg Med 1998 32 26 32 10.1016/S0196-0644(98)70095-2 9656945

[B2] Berlac P Hyldmo PK Kongstad P Kurola J Nakstad AR Sandberg M Scandinavian Society for Anesthesiology and Intensive Care Medicine Pre-hospital airway management: guidelines from a task force from the Scandinavian Society for Anaesthesiology and Intensive Care Medicine Acta Anaesthesiol Scand 2008 52 897 907 10.1111/j.1399-6576.2008.01673.x 18702752

[B3] Cummins RO Austin D Graves JR Litwin PE Pierce J Ventilation skills of emergency medical technicians: a teaching challenge for emergency medicine Ann Emerg Med 1986 15 1187 1192 10.1016/S0196-0644(86)80863-0 3752650

[B4] Tiah L Wong E Chen MF Sadarangani SP Should there be a change in the teaching of airway management in the medical school curriculum? Resuscitation 2005 64 87 91 10.1016/j.resuscitation.2004.07.011 15629560

[B5] Asai T Shingu K The laryngeal tube Br J Anaesth 2005 95 729 736 10.1093/bja/aei269 16286348

[B6] Ocker H Wenzel V Schmucker P Steinfath M Dörges V A comparison of the laryngeal tube with the laryngeal mask airway during routine surgical procedures Anesth Analg 2002 95 1094 1097 12351302 10.1097/00000539-200210000-00057

[B7] Wrobel M Grundmann U Wilhelm W Wagner S Larsen R Laryngeal tube versus laryngeal mask airway in anaesthetised non-paralysed patients A comparison of handling and postoperative morbidity Anaesthesist 2004 53 702 708 10.1007/s00101-004-0697-x 15167948

[B8] Kurola J Harve H Kettunen T Laakso JP Gorski J Paakkonen H Silfvast T Airway management in cardiac arrest--comparison of the laryngeal tube, tracheal intubation and bag-valve mask ventilation in emergency medical training Resuscitation 2004 61 149 153 10.1016/j.resuscitation.2004.01.014 15135191

[B9] Schalk R Meininger D Ruesseler M Oberndörfer D Walcher F Zacharowski K Latasch L Byhahn C Emergency airway management in trauma patients using laryngeal tube suction Prehosp Emerg Care 2011 15 347 350 10.3109/10903127.2011.561405 21521037

[B10] Wiese CH Semmel T Müller JU Bahr J Ocker H Graf BM The use of the laryngeal tube disposable (LT-D) by paramedics during out-of-hospital resuscitation-an observational study concerning ERC guidelines 2005 Resuscitation 2009 80 194 198 10.1016/j.resuscitation.2008.08.023 19010582

[B11] Schalk R Byhahn C Fausel F Egner A Oberndörfer D Walcher F Latasch L Out-of-hospital airway management by paramedics and emergency physicians using laryngeal tubes Resuscitation 2010 81 323 326 10.1016/j.resuscitation.2009.11.007 20006418

[B12] Stiell IG Wells GA Field B Spaite DW Nesbitt LP De Maio VJ Nichol G Cousineau D Blackburn J Munkley D Luinstra-Toohey L Campeau T Dagnone E Lyver M Ontario Prehospital Advanced Life Support Study Group Advanced cardiac life support in out-of-hospital cardiac arrest N Engl J Med 2004 12 647 656 10.1056/NEJMoa040325 15306666

[B13] Ochs M Davis D Hoyt D Bailey D Marshall L Rosen P Paramedic-performed rapid sequence intubation of patients with severe head injuries Ann Emerg Med 2002 40 168 171 10.1067/mem.2002.126370 12140494

[B14] Mort TC Emergency tracheal intubation: Complications associated with repeated laryngoskopic attempts Anesth Analg 2004 99 607 613 15271750 10.1213/01.ANE.0000122825.04923.15

[B15] Wiese CH Bartels U Bergmann A Bergmann I Bahr J Graf BM Using a laryngeal tube during cardiac arrest reduces "no flow time" in a manikin study: a comparison between laryngeal tube and endotracheal tube Wien Klin Wochenschr 2008 120 217 223 10.1007/s00508-008-0953-1 18500596

[B16] Ruetzler K Roessler B Potura L Priemayr A Robak O Schuster E Frass M Performance and skill retention of intubation by paramedics using seven different airway devices--a manikin study Resuscitation 2011 82 593 597 10.1016/j.resuscitation.2011.01.008 21353364

[B17] Nakstad AR Sandberg M Airway management in simulated restricted access to a patient--can manikin-based studies provide relevant data? Scand J Trauma Resusc Emerg Med 2011 19 36 10.1186/1757-7241-19-36 21668944 PMC3125355

